# Alphavirus Nucleocapsid Packaging and Assembly

**DOI:** 10.3390/v10030138

**Published:** 2018-03-20

**Authors:** Adriano Mendes, Richard J. Kuhn

**Affiliations:** 1 Department of Biological Sciences, Purdue University, West Lafayette, IN 47907, USA; mendes@purdue.edu; 2 Purdue Institute of Inflammation, Immunology and Infectious Disease, Purdue University, West Lafayette, IN 47906, USA

**Keywords:** alphavirus, assembly, RNA packaging, capsid protein, packaging signal

## Abstract

Alphavirus nucleocapsids are assembled in the cytoplasm of infected cells from 240 copies of the capsid protein and the approximately 11 kb positive strand genomic RNA. However, the challenge of how the capsid specifically selects its RNA package and assembles around it has remained an elusive one to solve. In this review, we will summarize what is known about the alphavirus capsid protein, the packaging signal, and their roles in the mechanism of packaging and assembly. We will review the discovery of the packaging signal and how there is as much evidence for, as well as against, its requirement to specify packaging of the genomic RNA. Finally, we will compare this model with those of other viral systems including particular reference to a relatively new idea of RNA packaging based on the presence of multiple minimal packaging signals throughout the genome known as the two stage mechanism. This review will provide a basis for further investigating the fundamental ways of how RNA viruses are able to select their own cargo from the relative chaos that is the cytoplasm.

## 1. Introduction

The genus *Alphavirus* is one of two genera within the family *Togaviridae*. Alphaviruses are ubiquitous small enveloped RNA viruses [1]. They have been identified in many different host species from birds to fish and mammals. Alphaviruses are arboviruses, which means they utilize arthropods as a vector between host species [2]. These viruses are primarily transmitted via hematophagous insects such as the mosquito (particularly those in the genus *Aedes*). There are currently 31 distinctive members, which are unofficially categorized as either old world or new world depending on geographic identification [3]. Although not a rule, old world viruses cause primarily an arthritic pathology while the new world viruses have been associated with a higher probability of encephalitis [4,5]. Despite nearly 40 years of research and many fundamental discoveries in virology, there are no licensed alphavirus vaccines or alphavirus specific antivirals. This has become a more serious concern given the recent outbreaks of Chikungunya (CHIKV) and Mayaro (MAYV) viruses as well as the increasing disease burden of Ross River virus (RRV) in Australia [6,7,8]. In this review, the topic of packaging and assembly of alphavirus particles will be examined. 

**Figure 1:**
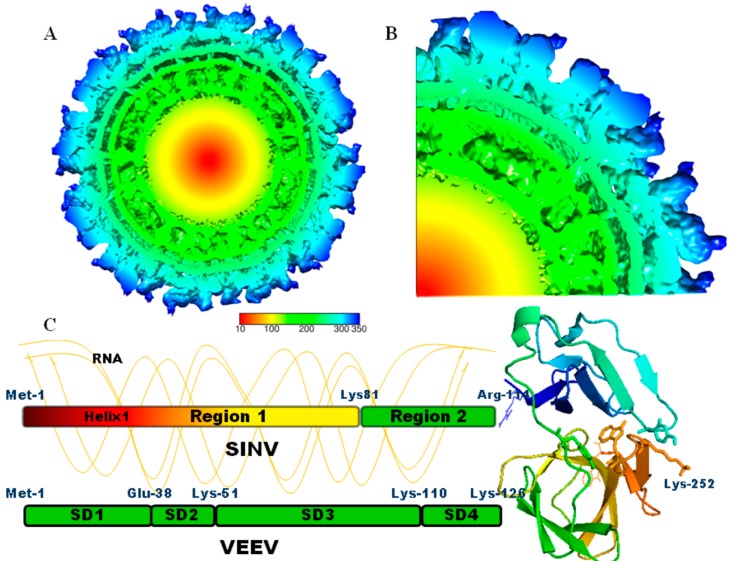
Structure of SINV and its CP: (**A**) Cryo-electron microscopy reconstruction of SINV, using the coordinates provided in Mukhopadhyay et al., 2009 [15]. The cross section is radially colored: center (red) to the outer surface (blue). The glycoprotein spikes (blue) are embedded in a lipid envelope (aqua to green). The NC is made up of CPs (green and yellow) and RNA (yellow and red). The scale bar is measured in Angstroms; (**B**) A wedged section of (**A**) has been enhanced depicting the glycoprotein envelope and the layers of density ascribed to the CP and the central RNA core. The C-terminal domain of the CP, shown in (**C**), could be fitted to the outer surface of the NC. The N-terminal domain together with the RNA is disordered; (**C**) Crystal structure (PDB: 1WYK) of the C-terminal protease domain. N-terminal and C-terminal residues, Arg114 and Trp264, are depicted as sticks. Shown in ball and stick are Tyr162, Tyr180, and Lys252, which make up the CP hydrophobic pocket, interacts with cdE2. The protein has been orientated so that the Arg114 is linked to the cartoon depiction of the N-terminal domain. Region I (Met1-Lys81) is highly positively charged and contains a single region of putative secondary structure known as helix I. Region II (Lys81-Arg114) was shown to be involved in selection of the genomic RNA. Depicted below SINV is the VEEV N-terminal domain described by the Frolov group.

In order to effectively understand the assembly of these viruses, one must first appreciate the structure of the particle. Multiple alphavirus structures have been reported using both X-ray crystallography and cryo-electron microscopy [9,10,11,12,13,14,15,16,17,18]. Alphavirus virions are approximately 70 nm in diameter and are made up of two concentric protein shells with *T* = 4 icosahedral symmetry (see Figure 1) [19]. The outermost shell is made up of the lipid envelope into which 240 copies of the E1/E2 glycoproteins are inserted. E1 and E2 appear on the surface as a trimer of heterodimers and give the particle its characteristic spiky surface representation [20,21]. The frame-shift product TF is also incorporated onto the virion surface [22,23,24]. Underneath the glycoprotein envelope and in contact with the C-terminus of E2 (cdE2), is the nucleocapsid core (NC), made up of 240 copies of the capsid protein (CP) and the genomic RNA [25,26]. Pentameric and hexameric arrangements of CPs make up the NC and are referred to as capsomers within the *T* = 4 NC structure [10]. It is unclear whether the 11 kb single stranded RNA genome assumes a single specific structure within the NC (see Figure 1). The first stage of NC assembly is the association between the CP and the RNA genome. This also defines the process of packaging since no empty cores have been identified during the alphavirus infection, which suggests that RNA plays an active role in the assembly process. RNA appears to be required for the process since in vitro assembly of NC, although promiscuous, does not continue without some form of nucleic acid [27]. It is therefore difficult to make a clear distinction between packaging and assembly since interaction with an RNA molecule serves to make the particle itself. In addition, it has been shown in multiple studies that many different packaging substrates can serve to create a NC particle. Furthermore, it has been difficult to derive an assembly model since very few NC intermediates have been reported. This review will focus on packaging and assembly of the NC and make little mention of how the core derives, its lipid envelope and glycoproteins as this topic will be covered by Margaret Kielian and co-workers in a related review.

## 2. The Capsid Protein

A single capped polyadenylated RNA, termed the 49S RNA, which refers to its sedimentation value, serves as the genome for these viruses. When introduced into the cell, the 49S RNA is translated into a p270 polyprotein, which codes for the non-structural proteins, nsP1–4 [1]. The structural proteins are translated from a subgenomic 26S RNA as a separate polyprotein (p130) [28]. The subgenomic RNA is also capped and polyadenylated and is derived but not replicated from the 49S genomic RNA. The CP is located at the N-terminus of p130. The CP of Sindbis virus (SINV), the prototype species of the genus, consists of 264 amino acids with a mass of approximately 30 kDa [29]. Upon translation of the p130 polyprotein, the CP is automatically proteolytically cleaved, which releases it into the cytoplasm. This exposes a signal sequence, which allows the remainder of the structural polyprotein (E3-E2-6K/TF-E1) to be inserted into the ER where it will undergo processing to form the components found in the viral envelope [30,31]. 

The CP can be separated into two domains including the highly positively charged N-terminal domain and the C-terminal protease domain with the latter forming the pentameric and hexameric capsomers of the NC [32,33]. The structure of the C-terminal domain (amino acids 114–264) of SINV revealed that it is a chymotrypsin-like serine protease. This structure also revealed that the enzymatic action only exists for a single self-cleavage event, which releases it from the polyprotein. This is because the C-terminal Trp-264 is inserted into the active site, blocking further function [25]. The N-terminal domain of the SINV CP has been characterized into two regions. Region I (amino acids 1–81) is highly positively charged and contains a putative secondary structure known as helix I (see Figure 1 and Table 1) [34]. Helix I is postulated to be involved in dimerization of CPs through the formation of coiled coil interactions [35]. The dimerization domain of the yeast protein GCN4 was shown to be a functional substitute for helix 1 in SINV [36]. Region II is a smaller stretch of amino acids (81–114) whose primary role is interaction with the RNA packaging signal (PS) [37,38]. A smaller amino acid sequence (99–114) at the C-terminus of region II is highly conserved among alphaviruses. In the crystal structure, the N-terminus (regions I and II) is disordered and unresolved [25]. A cryo-EM reconstruction of SINV was able to fit the C-terminal domain into the capsomer’s density on the surface of the NC [14,39]. Additional density that could not be fitted and did not assume any symmetry was ascribed to the N-terminal domain (see Figure 1). This suggests that the N-terminal domain of CP is flexible, largely disordered, and faces inwards towards the central RNA core. 

Recently, studies using VEEV have re-defined the N-terminal domain into four separate sub-domains called SD1–4 (Table 1) [40]. SD1 represents the N-terminal peptide of VEEV (amino acids 1–37) and SD2 (38–51), the location of a peptide which is similar to helix I in SINV. SD3 (52–110) represents the sequence with the greatest positive charge and is followed by SD4 (111–126), which is highly conserved among alphaviruses. These as well as previous studies from the same group have resulted in a model of assembly. This will be addressed later in this review. 

## 3. The Packaging Signal

Packaging signals can be defined as regions of RNA conserved in either sequence or structure, which enhance the specificity with which RNA is encapsulated. These signals are seldom essential for the assembly of the particle but can be an important determinant of whether assembly proceeds efficiently. Examples of defined packaging signals can be found in the genomes of HIV [49], Influenza virus [50], and the murine coronavirus mouse hepatitis virus [51]. In each case, these viruses require selective packaging due to the fact that more than one viral RNA species is used during infection. The same is true for the alphaviruses since it was noted that particles packaged exclusively 49S genomic RNA and not the 26S subgenomic RNA [52]. It therefore followed that the packaging signal (PS) must be present within the 49S and not 26S region of the genome. Identification of the PS was first defined from the study of defective interfering (DI) RNAs. Using a sequenced SINV DI RNA (DI-25), Weiss et al., 1989 [52] was able to show that a region between nucleotides 746 and 1226 in the nsP1 coding region on the genomic 49S RNA of SINV, was sufficient to allow a non-viral RNA to bind to the CP above the background level. This same sequence was used in gel-shift assays using SINV CP to demonstrate that the N-terminal domain could bind RNA [41]. Additionally, the sequence from nucleotides 945 to 1076 (nsP1 ORF) when cloned into a subgenomic RNA on SINV increased the packaging of the resulting RNA into particles [53]. 

An interesting aspect of the alphavirus infection is that one can use two separate RNAs to achieve infection of a host cell. Using this approach, a replicon RNA, which is able to self-replicate drives the amplification of a helper RNA that produces the structural proteins. Such systems have been harnessed for the over-production of heterologous proteins, development of vaccine strategies, and the identification of signals critical for replication and assembly [54,55,56,57,58]. It has been consistently observed that the addition of nucleotides 945–1076 to helper RNAs improves their packaging into particles [53,59,60]. However, helpers without this sequence are also able to package. The system is consistent with non-specific interactions of the N-terminal domain of the CP with RNA and the observation that not all DI RNAs retain the PS. Interestingly, a cellular tRNA^asp^ sequence can also allow for both replication and packaging of DI and helper RNAs, which is an observation that is still not properly understood [55,59,61]. Arguably the most detailed study of alphaviral PSs comes from Kim et al., 2011 [57]. In this report, a previously uncharacterized region of sequence conservation was identified in the nsP1 coding sequence between SINV, Venezuelan, Eastern, and Western equine encephalitis viruses (VEEV, EEEV and WEEV). This region from nucleotides 753–1116 (on SINV) corresponded to what previous reports had referred to as the PS. mFold predictions predicted a series of eight stem loops within the sequence with each containing triplet guanine (GGG) nucleotides at the stem tips. The overall secondary structure of the RNA, the GGG motifs and the number of loops containing GGG, which all had an effect on packaging specificity. Abrogation of the structure while maintaining the sequence resulted in an increase in subgenomic RNA packaging. Finally, this group was able to provide evidence for a long held hypothesis that the Semliki forest virus (SFV) clade of viruses has a PS in a different region of the genome. They were able to identify a similar structure in the nsP2 coding region of CHIKV, O’nyong-nyong (ONNV), MAYV, SFV, RRV and Getah virus (GV). This hypothesis originated from studies on SFV and RRV DI RNAs, which showed that similar sequences within the nsP2 gene when removed from DI genomes decreased their packaging into particles [53,62,63]. Therefore, a unique observation can be made of alphaviruses in that the PS may have evolved separately amongst different clades [57]. The balance of evidence thus suggests that in alphaviruses, the PS is responsible for a strong interaction with region II (SD4 in VEEV) on the N-terminal domain of the CP [37,52]. However, NC assembly does not necessarily depend on the PS since electrostatic interactions of the rest of the N-terminal domain also contribute to assembly. This has been shown experimentally using PS defective viruses which were still able to grow and helper as well as DI RNAs that were still packaged without the PS [53,56,57]. 

## 4. An in Vitro Model of NC Assembly

A fundamental way to study any complex system is to reduce it into its constituent parts and reassemble it. Accordingly, an in vitro NC assembly assay was created by Wengler et al. in 1982 [64] and was further developed by Tellinghuisen et al. in 1999 [65]. The resulting particles are termed core-like particles (CLPs) since they retain critical characteristics such as density, diameter, and overall morphology with natively purified NCs [26,64,65]. These studies have resulted in a number of critical observations about the mechanism of NC assembly. There is an absolute requirement for nucleic acid in oligomerization and assembly. This is consistent with previous observations that no empty alphavirus particles have been isolated. The nucleic acid does not have to be viral or RNA. From the 15 substrates tested in the original study, ssDNA of 6 and 12 nt as well as all dsDNAs were the only ones that failed to assemble CLPs. It therefore follows that charge neutralization is an important feature of the assembly mechanism and the secondary structure of the RNA is not important under in vitro conditions. There is an optimal ratio between nucleic acid and CP. This suggests, and logically so, that stoichiometry is a critical aspect of the assembly of 240 CPs and nucleic acid. By altering the size of the oligonucleotide used in the reaction we hypothesized that a head-full complement (a term adapted from bacteriophage biology) of nucleic acid is required to assemble a minimum NC. The N-terminal domain binds nucleic acid and may initiate assembly. Only limited truncation of this domain, up to 19 amino acids, was tolerated in the CLP experiments, which suggests a strict role for the majority of this domain in vitro. Lastly, dimers of CP are likely to initiate the assembly process. Using cross-linkers as well as a mutation that limited dimer formation (L52D), it was established that a CP-CP unit could initiate assembly and could substitute for non-viable CPs [27,66]. Therefore, the in vitro based assembly model postulates that the N terminal domain is responsible for both dimerization and RNA binding. CP may utilize the PS on the genome as a scaffold to bind region II (SD4 in VEEV). Concomitant or closely followed with this process is the interaction between CP dimers mediated by helix 1 (SD2) [35,38,66]. These interactions may serve to stabilize the flexible N-terminal domain and subsequently allow multimerization of further CPs into capsomers of which C-terminal domain interactions are most likely important. Capsomers then assemble into a stable NC. One would thus predict that intermediates such as dimers, pentamers, or hexamers could be purified from infected cells, which are analogous to the work done on picornavirus morphogenesis [67]. To date, no in vivo assembly intermediates have been isolated. It has been observed that when NCs are micro-injected into cells expressing envelope proteins, the NCs bud as if derived intracellularly [68]. This suggested at the time that NC assembly was the first step in the egress pathway, which ended in the budding of virus particles from the plasma membrane.

CHIKV virus-like particles(VLPs) have been generated from the heterologous expression of the structural proteins in both insect and mammalian cells [69,70,71]. These particles have been used in a Phase 1 clinical trial as a vaccine for CHIKV owing to the fact that the particles resemble wt particles and therefore illicit a relevant immune response [72]. Since these particles resemble wt particles but are not produced with replicating genomic RNA, an interesting question arises as to what RNA is packaged. Either cellular RNA or the transcript from which the VLPs are generated is assumed to be packaged, as recent cryo-EM reconstructions have revealed that the particles were not empty [73]. Unfortunately, we are unaware of published experimental data regarding the RNA inside these VLPs. Therefore, at face value, VLP studies appear to support in vitro evidence, which suggests that any RNA species can form NC particles. However, more work is needed in this area.

## 5. Models of Intracellular NC Assembly

Following their identification of the VEEV PS, Frolov and co-workers sought to characterize the mechanism of packaging in cell culture by extensively mutating the N-terminus of the CP. One of the most compelling results was that mutagenesis of all the positively charged amino acids still resulted in virus particle formation, albeit ones that packaged predominantly subgenomic RNA. These particles were termed pseudoinfectious viruses (PIVs) owing to their dramatically decreased infectivity [44]. Upon passaging these viruses, non-positively charged reversions resulted, which congregated on region II /SD4 of the N-terminal domain [44]. This highlights the importance of this highly conserved region as mutations in SINV at this site, which also affected the specificity of packaging [42]. Interestingly, similar mutations in SFV produced wt particles but prevented pre-formation of NCs in the cytoplasm [48]. According to the most recent model of assembly, SD1, 2, and 4 are involved in CP-RNA interactions that promote specificity. Therefore, other than the previously characterized helix 1 (SD2) and the conserved domain (SD4), this study presented the first pieces of evidence that the very N-terminus of CP (SD1) also regulates specificity [40]. In these studies, SD1 mutants were the most defective at forming PIVs. This data is consistent with the in vitro SINV assembly data in which truncations of more than 19 nt at the N-terminus were unable to assemble CLPs [65]. In a recent study, the sequence in SD1 was both randomized as well as mutated into EEEV and CHIK SD1 sequences. Compensatory mutations resulted in SD1 and SD2 from the passaging of these viruses, which was interpreted together with structural data from Zhang et al., 2011 [13], to suggest that SD1 and SD2 synergistically form a central core interaction critical for NC assembly [43]. SD3 was described as a negative regulator of assembly. This is because its mutation did not affect the specificity of RNA packaging despite being the most positively charged of the sub-domains. Therefore, SD3 was hypothesized to regulate the process by only allowing assembly when the charge in this domain was neutralized [40]. Reversions in nsP2 from SD2 mutant viruses also suggested a role for this sub-domain in binding to the replication complex. 

Overall, these results support the hypothesis that the positively charged N-terminus is not the only determinant of packaging. This domain does however form critical interactions for selecting the viral RNA and then proceeding with its assembly. The data suggests that adjacent C-terminal interactions can be sufficient to result in assembly in a cellular context even if the positive charge of the N-terminus is negated. They also suggest that location in the cell may play a role since subgenomic and not cellular RNA was packaged in many of the mutants described above. This was subsequently addressed in a paper in which revertants to the PIV particles were characterized. A series of reversions occurred in the replication protein nsP2, which improved viral fitness and suggested that nsP2 may influence RNA packaging [74]. The observation that elements of the replication complex influence packaging is supported by two other studies. The first showed that expression of nsP1–3 gave a packaging advantage to VEEV DI RNAs [56]. The second used a modified SINV with a tetracysteine tag to track the CP through infection. In this study, CP was associated with mobile puncta that co-localized with E2, nsP3, and the cellular stress factor G3BP [75]. Interestingly, pull down experiments have shown that G3BP is consistently found with replication complexes of alphaviruses [76,77]. However, experiments have not been conducted showing a direct effect on packaging specificity when only replication proteins are mutated. Therefore, it is possible that nsP2 could play the role of presentation of nascent RNA to CPs but the evidence is still inconclusive and further investigation is required. Alphaviruses like many RNA viruses alter intracellular membranes in order to create replication factories [78,79,80,81]. These alterations are collectively referred to as cytopathic vacuoles (CPVs). Type I CPVs are made up of a series of membrane invaginations known as spherules. These are derived from endosomal membranes and are believed to be the site of alphavirus RNA synthesis [82,83]. The involvement of nsP2 in a packaging model would place the location of NC packaging and assembly at the replication complex, which may be an additional means to improve the specificity of packaging. When individual CP subdomains (SD1–4) in VEEV were deleted and introduced into BHK cells, SD1, 3, and 4 deleted viruses produced VLPs. These VLPs packaged a greater proportion of actin mRNA, which suggests that the location of RNA packaging was disrupted in these mutants [40]. The idea that the site of NC assembly is proximal to the replication complex would also match a well described feature of alphavirus infection when observed by electron microscopy in that NCs accumulate in the cytoplasm prior to budding [84,85]. 

For a long time, the pre-formation of NCs in the cytoplasm was an assumed feature of the assembly process. However, various lines of evidence suggest that alphaviruses can also follow a pathway in which budding and assembly occurs simultaneously. This was first revealed via mutations to the conserved domain of SFV (see Table 1, SD4 in VEEV and region II in SINV). The data indicated that although particle production appeared wt, NCs accumulated at the plasma membrane instead of the cytoplasm via electron microscopy [48]. The same group later showed using cryo-EM that when much of the N-terminal domain (amino acids 40–118) was removed from SFV, the particles retained the structural characteristics of a wt particle. The mutant particles were however less stable and packaged both genomic and subgenomic RNA [86]. These studies were the first to argue against NC pre-formation and that the glycoprotein spikes were sufficient to nucleate RNA-CP complexes at the plasma membrane and thus bud virus. When mutations were made in the proposed binding site between CP and cdE2, morphological variants of SINV were generated with alternative triangulation numbers [87]. The same group also showed that by mutating SINV using additional Furin cleavage sites in E1 and E2, helical, non-icosahedral virus particles were produced [88]. This data supports the notion that interactions between glycoproteins can influence the NC and may present a variation of the alternative pathway of simultaneous assembly at the PM. The SD mutant VEEVs discussed earlier support the SFV data and showed similar phenotypes of subgenomic RNA packaging and particle instability when most of the positively charged amino acids of the N-terminus of CP were mutated [40,44]. These studies prompted this group to add to their generalized assembly model (mentioned above) that in the absence of pre-formed NCs, alphavirus also possess a pathway that allows CP-RNA complexes to bud directly from the plasma membrane nucleated by the glycoproteins. In addition, there is the possibility that the CP could derive its RNA at this location since replication spherules originate at the PM [82]. It is thus possible that alphaviruses are able to replicate, assemble NC, and bud at the PM. 

**Figure 2:**
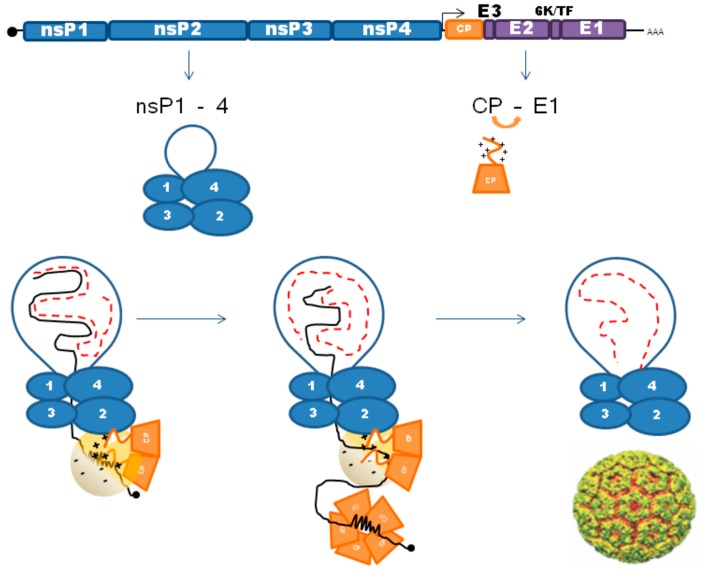
Model of NC assembly: Upon entry into the cell the alphavirus genome RNA is initially translated into the replication proteins nsP1-4. A spherule results when the replication complex of nsP1–4 (1–4) begins to synthesize RNA. RNA synthesis utilizes a negative strand (red dashed line) intermediate to make more genomic RNA (solid black line). As the replication complex matures, the subgenomic promoter is favored resulting in the structural polyprotein CP to E1. The protease activity of CP on its C-terminal domain cleaves it off nascent polyproteins co-translationally. Evidence thus far suggests that dimeric CP initiates assembly via multiple interactions with its positively charged N-terminal domain and the newly synthesized RNA. This process may be mediated by specific interactions between SD4 and the PS on the genome and a potential interaction between CP and the replication complex (nsP2). Assembly beyond this point is largely a mystery as there is yet to be any evidence of intermediate states. Comparison to the multiple packaging signal hypothesis of Stockley and colleagues suggests that electrostatics play a comparatively more prominent role in alphavirus assembly. However, this does not rule out the theory that RNA compaction could be involved. NC cores accumulate in the cytoplasm and can be visualized as discrete entities at sites distal to replication spherules.

In summary, both in vivo and in vitro studies have thus far concluded that NC assembly is not a reaction simply mediated by a CP-PS interaction (see Figure 2). The subdomains in the N-terminal domain appear to govern many different reactions from selecting the RNA and binding adjacent CP molecules as well as sending the protein to the correct intracellular location. Both specific and non-specific interactions are necessary in this regard. The C-terminal protease domain is not passive in the assembly process since it governs the geometry and symmetry of the particle but can also drive assembly when the N-terminal domain is mutated significantly. An additional determinant may be the location of NC assembly since there is evidence that the replication complex may interact with CPs. However, more work needs to be done to substantiate this hypothesis.

## 6. The Two Stage Assembly Mechanism

**Figure 3:**
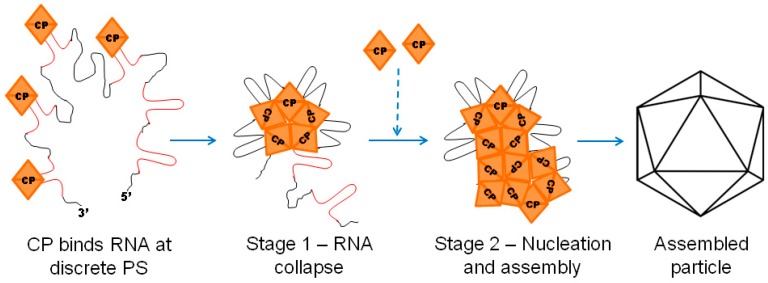
Model of the two stage assembly mechanism: The model is based off of evidence that multiple discrete PS exist on the genomes of viruses such as MS2, STNV, and HBV. These PS (highlighted in red) serve as binding sites for the CP of the virus. Stage 1 refers to the collapse of the RNA into a condensed form following inter-CP interactions. Stage 2 is represented by nucleation of the CPs on the RNA and the addition of further CP molecules (dotted arrow), which result in assembly of the particle. In this manner, multiple PS, dispersed on the genome, define the incorporation of the CP and the eventual assembly of the particle. Adapted from Borodavka et al., 2012 [89].

Recently, Peter Stockley and Reidun Twarock of the Universities of York and Leeds, respectively, have developed an alternative theory for RNA packaging and assembly. This theory is called the two stage mechanism of viral compaction or the packaging signal hypothesis [89,90]. It states that the first stage of assembly of a single stranded RNA virus is RNA compaction (see Figure 3). Using single molecule fluorescence spectroscopy (smFCS) on in vitro reassembly reactions of the bacteriophage MS2 and the plant virus Satellite Tobacco Necrosis Virus (STNV), they were able to show that labelled RNA molecules had a larger hydrodynamic radius than the space available within the NCs. When CPs of the relevant viruses were added into the reactions, the RNA radii decreased, which suggests that the RNA had compacted to fit into the NC. They go on to describe the second stage as the assembly stage in which further multimers of CP co-operatively follow the physical path laid out by the RNA to form fully assembled particles [91]. This hypothesis shifts away from traditional capsid-centric based assembly ideas towards an RNA-centric one (see Figure 3). It stems from earlier work in which the structure of the MS2 particle revealed a distinctive lattice of density attributed to the RNA genome beneath that of the capsid [92]. This density was subsequently mathematically modelled using Hamiltonian paths. This model describes a continuous and energetically favorable pathway that the RNA could adopt in which it makes contacts with the capsid at sites that were predicted in the cryo-EM reconstruction [93]. The symmetry of the particle is thus defined by the RNAs interaction and effect on the CP. In recent work, this group has used systematic evolution of ligands by exponential enrichment (SELEX) to identify multiple PSs along the genome, which would act as sites upon which compacted RNA could interact with CPs and regulate the assembly process. These PSs are believed to have a direct influence on the CP, depending on the interaction, which allows its assembly into a NC [91,94,95]. The hypothesis can be compared to stapling together proteins from the inside. Each PS acts as a staple on a path, which influences the path itself as well as future CP-CP interactions. The most accurate structure of MS2 was published in January 2017 by a group not associated with this hypothesis [96]. There has yet to be any formal comparison between the RNA interactions identified in the new structure and the predictions made in previous studies.

## 7. Could Alphaviruses Assemble Using the Two Stage Mechanism?

Evidence for the two stage model has been described for MS2, STNV, Hepatitis B virus (HBV), Hepatitis C virus (HCV), and members of the *Picornaviridae* [91,94,95,97,98]. Therefore, this mechanism may be considered as a general assembly paradigm and it is worth asking whether this applies to the alphaviruses. To the best of our knowledge, no studies have been done using in vitro assembled alphavirus NCs in smFCS experiments. This would provide critical evidence behind whether RNA compaction plays a role in alphaviruses. It is hard to imagine a scenario where RNA is not compacted to some degree during assembly. In the case of alphaviruses, the hypothesis is that this is driven by the electrostatic neutralization between RNA and the N-terminal domain of the CP. In viruses described in the two stage model, the suggestion is that the RNA provides the pathway and actively participates in making CPs competent for NC assembly. This therefore results in specific sequences inhabiting specific locations on the interior of the particle. Unlike in MS2, none of the reconstructions of alphavirus CLPs or wt NCs have revealed a lattice of RNA density. It is, therefore, fair to assume that the alphavirus RNA has a wider ensemble of states within the NC compared to two stage virions. The PSs identified for viruses of the two stage model are scattered throughout the genome with similar secondary structures. According to the theory, this is so that the RNA can maintain specific contacts with CPs, which are required for assembly to proceed along the pathway. There is no evidence that multiple similar secondary structural elements throughout the genome play any role in alphavirus assembly. This is also unlikely given that CLPs assembled with vastly different RNA or DNA substrates in vitro. The minimum size of a substrate required for in vitro assembly was found to be 14 nt. Therefore, it is unlikely that the CP could follow such a small nucleic acid sequence [65]. This suggests that electrostatic neutralization may be of greater importance in alphavirus assembly. Since a PS has already been identified, this signal may serve to nucleate CPs at the site of assembly, which allows electrostatic energy to drive the rest of the process. One means to align these theories could be to compare the alphavirus PS to the TR stem-loop in MS2. TR serves to nucleate the CP of MS2 and commence RNA collapse. If the alphaviral PS were to do the same, then the rest of the genome may be responsible for neutralizing the N-terminal domains in such a way that certain domains (SD1–4) make more important contacts than others. This would align the observations made by the Frolov group with a two stage-like mechanism. 

Therefore, the detailed molecular mechanisms describing alphavirus NC genome packaging and assembly remain elusive. Traditional approaches have identified some viruses with singular RNA secondary structures, described as a PS, which are able to nucleate CPs. This initiates an electrostatically driven assembly process. However, many viruses do not conform to this model as a single PS could not be identified. For these, the newer two stage theory appears more valid in that multiple PSs achieve assembly by guiding the capsid protein down a specific RNA packaging pathway. The real answer may lie in some combination of the two theories and further investigation into alphavirus assembly may be a useful system to investigate this. Given the recent advances in structure determination using cryo-EM, it may be worth revisiting the structure of the alphavirus NC and designing experiments to determine whether specific RNA sequences make contacts with the N-terminal domain. In a truly electrostatic mechanism, it is unlikely that a specific sequence would be necessary. 

## 8. Outstanding Questions and Conclusions

Additional questions about alphavirus assembly include investigating the site of NC assembly within the cellular context. The prevailing hypothesis is that assembly occurs in or near replication spherules as they are the most obvious place where CP could find genomic RNA. However, specific evidence to support this idea is lacking. The current evidence indicates that spherules begin biogenesis at the PM and then become CPVIs. A question is raised whether CPVIs are required for NC assembly or whether spherules at the PM play any role in the alternative assembly model from the PM. It is also important to determine how the CP or genome RNA traffics to sites of assembly. Unpublished data in our laboratory suggest that intermediate filaments provide a network on which NCs traffic between CPVIs and IIs. There is also a line of research dedicated to understanding the role of the CP upon entry into new cells. Evidence from Sokoloski et al., 2017, suggests that specific CP-RNA interactions generated during assembly or upon disassembly can alter the kinetics of viral growth in a subsequent cell [99]. More directly related to the mechanism of assembly and perhaps the most important task is to provide reliable evidence for assembly intermediates. Many old and new theories rely heavily on the knowledge of at least one step between the first (monomer) and last (particle) species, information that is drastically lacking in the alphavirus literature. One of the major roadblocks in this regard has been the inability to track discreet CP states. One way to do so would be to derive dimer vs. monomer CP antibodies. This would greatly facilitate both the localization and trapping of further states. For many, the question of how a NC forms is a fundamental one. To some this is seen as a potential antiviral target independent from the host cell. Our view is that virology has always been a tool for understanding the basic building blocks of biology and we see that the molecular machinery of how viruses are created as another means for appreciating the world within a cell.

## Figures and Tables

**Table 1:** Description of the domains and regions associated with NC assembly of three commonly referenced alphaviruses.

Virus	Domain	Region/Sub-Domain	Amino Acids	Proposed Function	References
SINV	N-terminal		1–114	Initiating assembly and RNA specificity	[33]
		Region I	1–81	Specificity of RNA packaging	[38]
		Helix I	38–55	CP dimerization	[34,35,36]
		Region II—conserved sequence	81–114	Interaction with RNA PS	[37,41,42]
	C-terminal		114–264	Protease domain, interaction with cdE2	[14,25]
VEEV	N-terminal	SD1	1–38	Initiating assembly and RNA specificity	[40,43]
		SD2	39–51	CP dimerization, initiating assembly, binding replication complex and RNA specificity	[40,43]
		SD3	52–110	Negative regulator of assembly	[40]
		SD4—conserved sequence	111–126	Initiating assembly and RNA specificity	[40,44]
		Nuclear localization sequence	38–60	Inhibition of host cell transcription	[45,46]
	C-terminal		127–275	Protease domain, interaction with cdE2	[13]
SFV	N-terminal	RNA-binding region	1–104	Specificity of RNA interaction	[47,48]
		Conserved linking peptide	105–118	Pre-formation of NCs	[48]
	C-terminal		119–267	Protease domain	
